# Supplementary material for: Genome-Wide Association Study of Growth and Sex Traits Provides Insight into Heritable Mechanisms Underlying Growth Development of Macrobrachium nipponense (Oriental River Prawn)
Source: Biology (Basel). 2023 Mar 10;12(3):429. doi: 10.3390/biology12030429 (PMC10045025; doi:10.3390/biology12030429)
Supplement: Supplementary file 1 [file biology-12-00429-s001.zip › biology-1997912-supplementary.pdf]

Supplementary Materials:

Software resources

SAIGE(https://github.com/weizhouUMICH/SAIGE),

GEMMA (https://github.com/genetics-statistics/GEMMA)

R(https://www.rstudio.com/)

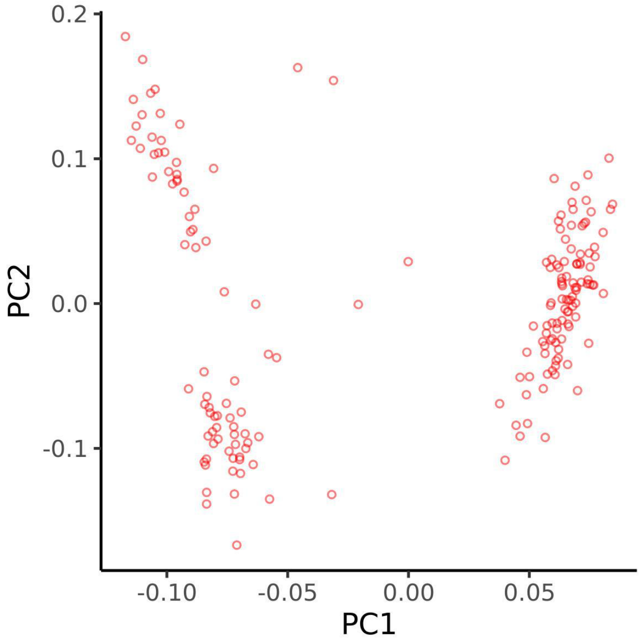

Figure S1. The Principal component analysis of population for *Macrobrachium nipponense*
